# Improved diagnostic performance of high‐sensitivity cardiac troponins in muscle dystrophies using comprehensive definition criteria for cardiac involvement: A longitudinal study on 35 patients

**DOI:** 10.1111/ene.16498

**Published:** 2024-09-30

**Authors:** Mustafa Yildirim, Christian Salbach, Christoph Reich, Regina Pribe‐Wolferts, Barbara Ruth Milles, Tobias Täger, Matthias Mueller‐Hennessen, Markus Weiler, Benjamin Meder, Norbert Frey, Evangelos Giannitsis

**Affiliations:** ^1^ Department of Internal Medicine III, Cardiology University Hospital of Heidelberg Heidelberg Germany; ^2^ Institute for Cardiomyopathies and Center for Cardiogenetics, Department of Medicine III University of Heidelberg Heidelberg Germany; ^3^ Department of Neurology University Hospital of Heidelberg Heidelberg Germany; ^4^ German Center for Cardiovascular Research Standort Heidelberg/Mannheim Heidelberg Germany; ^5^ Stanford Genome Technology Center Stanford University School of Medicine Palo Alto California USA

**Keywords:** cardiac involvement, cardiac troponin, diagnosis, muscular dystrophy, skeletal muscle dystrophies

## Abstract

**Background and purpose:**

Sparse information is available on the correct interpretation of elevated high‐sensitivity cardiac troponin (hs‐cTn) in confirmed muscular dystrophies.

**Methods:**

Serum concentrations of hs‐cTn T (hs‐cTnT) and hs‐cTn I (hs‐cTnI) were determined in 35 stable outpatients with confirmed skeletal muscle dystrophies. We calculated sensitivities, specificities, and positive and negative predictive values of hs‐cTnT and hs‐cTnI for identification of cardiac involvement using a comprehensive definition that included diastolic left ventricular and right ventricular function, strain analysis using two‐dimensional transthoracic echocardiogram and magnetic resonance imaging, myocardial biopsies, and consideration of a variety of triggers for cardiac injury, including arrhythmias, conduction disorders, and hypoxemia due to respiratory failure.

**Results:**

Cardiac involvement was diagnosed in 34 of 35 cases. Specificities of hs‐cTnT increased from 12.5% to 100% (*p* = 0.0006) applying the comprehensive definition compared to a definition based on electrocardiography and echocardiography alone. At the recommended 99th percentile upper limit of normal, sensitivities were significantly lower for hs‐cTnI than for hs‐cTnT (29.4% vs. 100%, *p* = 0.0164). Conversely, the specificities of hs‐cTnT and hs‐cTnI increased to 100% when using the comprehensive definition criteria for diagnosing cardiac involvement.

**Conclusions:**

Elevated hs‐cTnT but not hs‐cTnI discriminates cardiac involvement in cases with confirmed skeletal muscle dystrophies with very high sensitivity and 100% specificity. Prior reports on worse performance may be explained by the use of less sensitive imaging methods or incomplete assessment of cardiac involvement.

## INTRODUCTION

The prevalence of cardiac involvement among patients with skeletal muscle dystrophies varies across the range of different skeletal muscle dystrophies and due to the use of inappropriate or incomplete assessment of cardiac involvement [1, 2, 3, 4, 5]. A high prevalence of structural abnormalities of the heart ranging from 84% to 96% has been reported for Duchenne and Becker dystrophies [6, 7]. Other manifestations of cardiac involvement may include paroxysmal arrhythmias, for example, atrial fibrillation or atrial flutter, bradycardias, or conduction disorders, that are more difficult to document. Moreover, many patients with muscle dystrophies suffer from hypoxemia and recurrent lower respiratory tract infections from respiratory failure [4, 8]. The aforementioned complications that stipulate an elevation of high‐sensitivity cardiac troponin (hs‐cTn) may explain the observation of false positive cTn elevations in previous observational studies [9, 10, 11, 12, 13, 14, 15]. In addition, the discrepancy between elevated hs‐cTn and absence of overt structural abnormalities may be explained in part by low utilization rates of sensitive imaging methods, incomplete assessment of diastolic or right ventricle (RV) function, and length time bias due to earlier detection of cardiac involvement by cTn compared to imaging. Before the development of overt cardiomyopathy, localized hypertrophy, diffuse fibrosis, focal myocardial scarring, and fibrofatty replacement of myocardium may have already occurred but may only be visualized—if at all—using modern magnetic resonance imaging (MRI) techniques [16, 17].

Therefore, we studied the performance of hs‐cTn T (hs‐cTnT; Roche Diagnostics) and hs‐cTn I (hs‐cTnI; Siemens Atellica IM) to identify cardiac involvement in a cohort of stable patients with confirmed muscle dystrophies who were referred from the neurology department for diagnosis of cardiac involvement and/or monitoring of cardiac disease.

## METHODS

### Study design

We prospectively enrolled all patients with confirmed skeletal muscle dystrophies who were referred for diagnosis and collaborative management in an outpatient cardiology ward of the University Hospital of Heidelberg between 1 December 2021 and 31 December 2022.

### Data collection

A sample size calculation was not performed, because the number of eligible patients was small and because all cases were enrolled unless they refused consent. Information was collected on demographics, medical history, biopsy findings from skeletal muscle and cardiac tissue, electrocardiography (ECG), or imaging information from transthoracic echocardiography (TTE) including systolic and diastolic left ventricle (LV) function, RV function, and pulmonary artery pressure, and/or cardiac MRI (cMRI) with late gadolinium enhancement (LGE). Moreover, information was collected on the occurrence of atrial or ventricular (tachy‐)arrhythmias, conduction disorders, paced rhythm, prolonged episodes of hypo‐ or hypertension, or hypoxemia from respiratory failure.

Before referral to the cardiology clinic for a definitive evaluation of cardiac involvement, diagnoses were made by treating physicians from external clinics not involved in the present study. These initial diagnoses were primarily based on ECG, standard cardiac imaging modalities such as two‐dimensional (2D) TTE, and standard cMRI. For the present study, the presence or absence of a cardiac manifestation was readjudicated using advanced cardiac imaging including strain, diastolic LV and RV function, contrast‐enhanced cMRI, and additional definition criteria that included factors that are potentially linked to an elevation of hs‐cTnT or hs‐cTnI such as atrial fibrillation, episodes of hypo‐ or hypertension, and bouts of hypoxemia due to lung involvement.

The routine follow‐up interval was 6–12 months in person. The exclusion criteria solely comprised refusal to provide informed consent, refusal of venipuncture or blood sampling, missing or incomplete blood sampling, or withdrawal of consent.

### Blood sample collection and laboratory analysis

Blood samples were collected at the index visit and at follow‐up. Roche fifth generation hs‐cTnT (Roche Diagnostics) was measured on a COBAS E601, and Siemens hs‐cTnI (Atellica Immunoassay) and N‐terminal pro‐B‐type natriuretic peptide (NT‐pro BNP; Siemens Atellica IM NT‐proBNP) were measured along with routine blood chemistry from fresh plasma, and 20‐mL aliquots were stored at −80°C for additional biomarker measurements. The limit of blank and limit of detection were established as 2 and 3 ng/L for hs‐cTnT and 0.5 and 1.6 ng/L for hs‐cTnI [18, 19]. In our laboratory, the coefficient of variation was determined to be 10% at a concentration of 13 ng/L based on 100 measurements. Except for the second blood sample at the index outpatient visit, no additional tests were performed for study purposes. The study was approved by the local ethics committee of the University of Heidelberg, and the entire study was conducted in accordance with the 1964 Declaration of Helsinki. Patients provided informed consent for study participation and the additional blood sampling. The trial was registered on ClinicalTrials.gov (identifier: NCT05779202).

### Definitions

*Cardiac involvement* was determined through imaging that showed a left ventricular ejection fraction (LVEF) < 52%, following the guidelines of the American Society of Echocardiography (ASE) and the European Association of Cardiovascular Imaging (EACVI) [20]. Additionally, the definition encompassed the presence LGE on contrast‐enhanced cMRI, as well as morphological abnormalities of the RV or LV on TTE or cMRI. These abnormalities encompassed ventricular hypertrophy, dilatation, wall motion abnormalities, and structural defects like septal abnormalities or congenital anomalies. Evidence of diastolic dysfunction on 2D TTE was categorized into grades I–III, based on ASE and EACVI guidelines [21], although pathological myocardial biopsy findings were also considered. Imaging evidence of reduced LVEF was obtained either by cMRI or, if cMRI was not possible, by TTE. Additional criteria for cardiac involvement, in line with those in prior studies [22, 23], included abnormal ECG findings, rhythm disturbances such as supraventricular tachycardia and atrial fibrillation/flutter, conduction abnormalities, paced rhythm, cardiac arrest due to ventricular tachycardia or ventricular fibrillation (sudden cardiac death occurring within 1 h of acute symptoms), appropriate treatments by implantable cardioverter‐defibrillator like antitachycardia pacing or shock, arterial hypertension, and hypoxemia caused by respiratory failure.

*Lung involvement* was defined as the need for ventilatory support such as daily or nocturnal noninvasive ventilation, or a history of respiratory failure requiring intensive care unit care, as noted in previous studies [24, 25, 26].

### Follow‐up

The routine follow‐up interval was 6–12 months in person. At follow‐up, information was collected on the progression of cardiac involvement, incident complications, admission to intensive care units, escalation of supportive therapies, need for hospitalization, and length of hospital stay. No patients died. The exclusion criteria solely comprised refusal to provide informed consent, refusal of venipuncture or blood sampling, missing or incomplete blood sampling, or withdrawal of consent.

### Statistical analysis

Statistical methods included descriptive analysis of absolute and relative frequencies, comparison of clinical and prognostic parameters between groups using the chi‐squared test for categorical parameters, and the Student *t*‐ or Mann–Whitney *U*‐test for continuous variables. Sensitivities, specificities, and negative and positive predictive values were calculated as usual. We determined diagnostic performance for diagnosis of cardiac involvement from receiver operating characteristic (ROC) curves on the basis of the continuously measured hs‐cTnT and hs‐cTnI and compared areas under the curve (AUCs) using the test of DeLong et al. [27].

All statistical analyses were carried out using R software (version 4.3.0, R Foundation for Statistical Computing).

## RESULTS

### Patient characteristics

**FIGURE 1:**
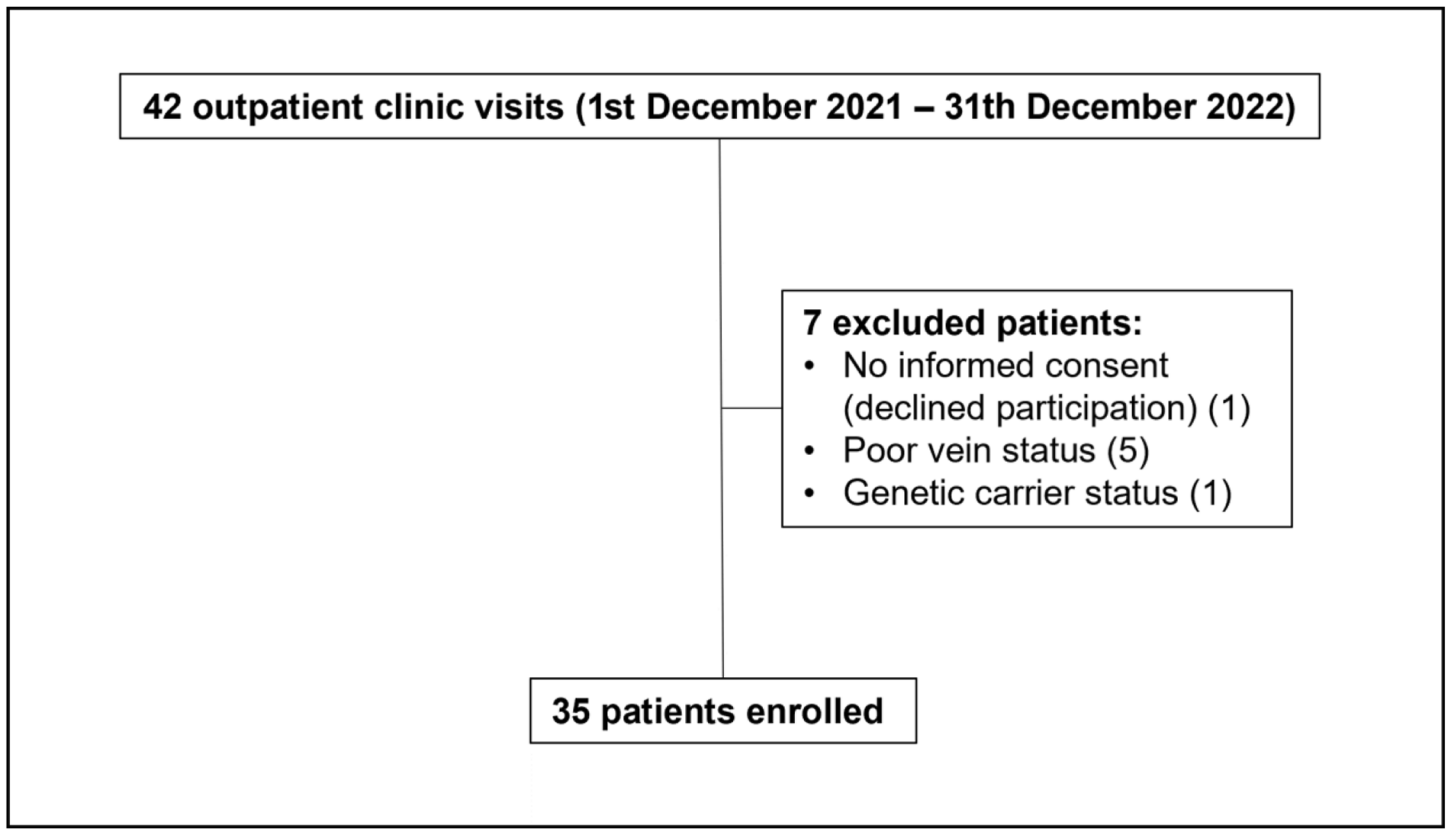
Flowchart for included and excluded patients. Flowchart depicts the process of patient inclusion and exclusion throughout the study. The chart details the total number of patients enrolled, as well as those excluded, with reasons for exclusion provided.

During the 12‐month enrollment period, a total of 35 patients were enrolled. Figure 1 presents the flow diagram of their inclusion and exclusion. All patients had been diagnosed with biopsy‐confirmed skeletal muscular dystrophy at the Department of Neurology of Heidelberg University Hospital or had been diagnosed by DNA analysis at the Cardiology Department of Heidelberg University Hospital. Before readjudication, cardiac involvement was diagnosed in 27 of 35 cases (77.1%).

Baseline characteristics are shown in Table 1. In brief, mean age was 37 years (interquartile range [IQR] = 23–50), and median body mass index was 23.9 kg/m^2^ (IQR = 20.6–27.7). Eight cases (23%) were female, 34 cases (97.1%) had cardiac involvement, and 10 (29%) had pulmonary involvement. Among the 35 patients, 14% (five cases) had a previous history of atrial fibrillation, 8.5% (three cases) had a history of atrial flutter, and 14% (five cases) presented with arterial hypertension. Additionally, 14% (five cases) of the cases showed hypercholesterolemia (Table 1).

**TABLE 1:** Baseline characteristics.

Characteristic	All patients, *N* = 35
Age, years (IQR)	37.4 (23–50)
Female sex, *n* (%)	8 (23)

Height, cm (IQR)	170.7 (168–179)
Weight, kg (IQR)	70.7 (57.5–85)
BMI, kg/m^2^ (IQR)	23.9 (20.6–27.7)
Heart rate, bpm (IQR)	71 (59.5–78)
Systolic pressure, mmHG (IQR)	106 (100–114)
Diastolic pressure, mmHG (IQR)	69 (60–75)
Follow‐up, months (IQR)	6.6 (6–7)
Laboratory	
hs‐cTnT 0 h, ng/L (IQR)	34.4 (17.5–46.2)
hs‐cTnT 0 h ≥ 14 ng/L, *n* (%)	33 (94.3)
hs‐cTnT 1 h, ng/L (IQR)	32.3 (17.2–42.9)
hs‐cTnT 1 h ≥ 14 ng/L, *n* (%)	34 (97.1)
hs‐cTnT FU, ng/L (IQR)	34.3 (16.8–43.9)
hs‐cTnT FU > 14 ng/L, *n* (%)	33 (94.3)
hs‐cTnI 0 h, ng/L (IQR)	20 (6–43)
hs‐cTnI 1 h, ng/L (IQR)	26 (7–41)
hs‐cTnI FU, ng/L (IQR)	17 (6–42)
hs‐cTnI 0 h, 1 h, or FU ≥ 45 ng/L, *n* (%)	10 (28.6)
Creatinine, mg/dL (IQR)	0.55 (0.18–0.8)
eGFR, mL/min/1.73 m^2^ (IQR)	158.6 (97.9–211.1)
Cystatin C, mg/L (IQR)	1.1 (0.9–1.17)
NT‐proBNP, ng/L (IQR)	359 (113–605)
CK, U/L (IQR)	501.3 (216.5–754)
Myoglobin, μg/L (IQR)	150 (84.5–190.5)
Diagnostic workup	
TTE LVEF, % (IQR)	45 (35–54.3)
TTE LVEF < 52%, *n* (%)	24 (69)
Diastolic dysfunction grade II, *n* (%)	11 (31.4)
Diastolic dysfunction grade III, *n* (%)	6 (17.1)
Conduction disorders, *n* (%)	11 (31.4)
cMRI LVEF, % (IQR)	49.2 (40–60) [*n* = 19]
Cardiac involvement, *n* (%)	34 (97.1)
Lung involvement, *n* (%)	10 (29)
Atrial fibrillation, *n* (%)	5 (14)
Atrial flutter, *n* (%)	3 (8.5)
Hypertension, *n* (%)	5 (14)
Hypercholesterolemia, *n* (%)	5 (14)
ICD, *n* (%)	2 (5.7)
Pacemaker, *n* (%)	2 (5.7)
Chronic kidney disease, *n* (%)	5 (14)
Medication distribution, *n* (%)	
ASS	3 (8.5)
NOAC	6 (17)
Phenprocoumon	1 (2.8)
ACE inhibitors	24 (68.6)
Sacubitril/valsartan	6 (17)
Beta‐blockers	28 (80)
ARB	11 (31.4)
Diuretics	8 (22.9)
SGLT2 inhibitors	11 (31.4)

Abbreviations: ACE, angiotensin‐converting enzyme; ARB, angiotensin receptor blockers; ASS, acetylsalicylic acid; BMI, body mass index; bpm, beats per minute; CK, creatine kinase; cMRT, cardiac magnetic resonance imaging; eGFR, estimated glomerular filtration rate; FU, follow‐up; hs‐cTnI, high‐sensitivity cardiac troponin I; hs‐cTnT, high‐sensitivity cardiac troponin T; ICD, implantable cardioverter‐defibrillator; IQR, interquartile range; LVEF, left ventricular ejection fraction; NOAC, non‐vitamin K antagonist anticoagulant; NT‐proBNP, N‐terminal pro‐B‐type natriuretic peptide; SGLT2; sodium–glucose cotransporter‐2; TTE, transthoracic echocardiography.

Moreover, a comprehensive overview of the medication distribution within the study cohort is also provided in Table 1, highlighting the prevalence of specific medications among the participants.

### Diagnostic methods

Twelve‐lead ECG and TTE were performed in all patients. The median LVEF was 45% (IQR = 35–54.3). cMRI was performed in 19 patients (54.3%). Among these patients, 10 (53%) individuals exhibited LGE. Myocardial biopsies were available in four (11.4%) patients. Diastolic dysfunction grade II was observed in 11 patients (31.4%), whereas grade III was present in six patients (17.1%), with no patients exhibiting grade I diastolic dysfunction. Arrhythmias were registered in 21 (60%) patients, including atrial fibrillation (14%), atrial flutter (8.5%), and miscellaneous others (37.1%). Conduction defects were observed in 11 patients (31.4%), with occurrences including atrioventricular blocks of varying degrees (17.1%), right or left bundle branch block (8.6%), and paced rhythm (5.7%).

**FIGURE 2:**
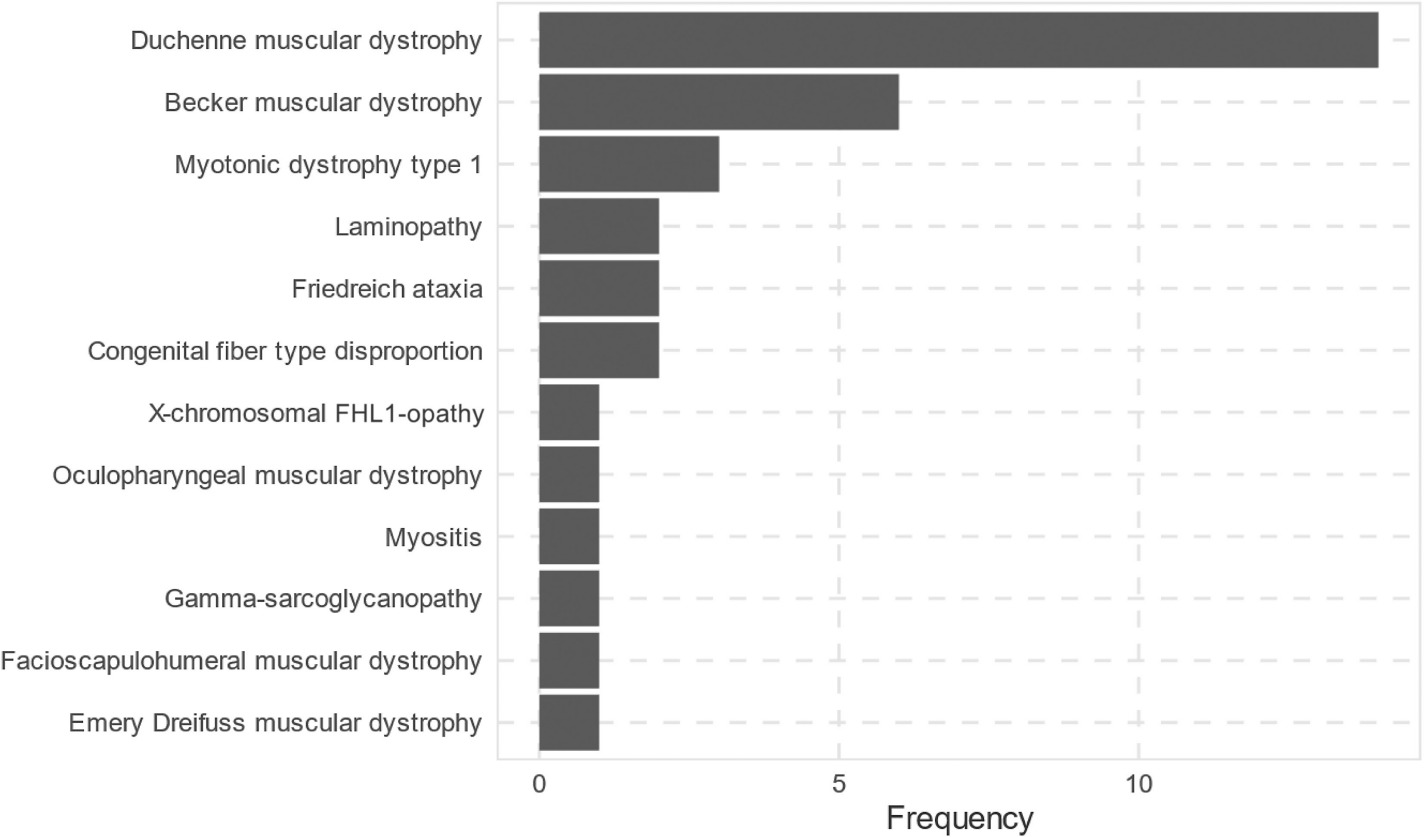
Distribution of muscular dystrophy subtypes in the study population.

The distribution of individual disease entities is depicted in Figure 2.

### Performance of hs‐cTnT and hs‐cTnI for diagnosis of cardiac involvement

**FIGURE 3:**
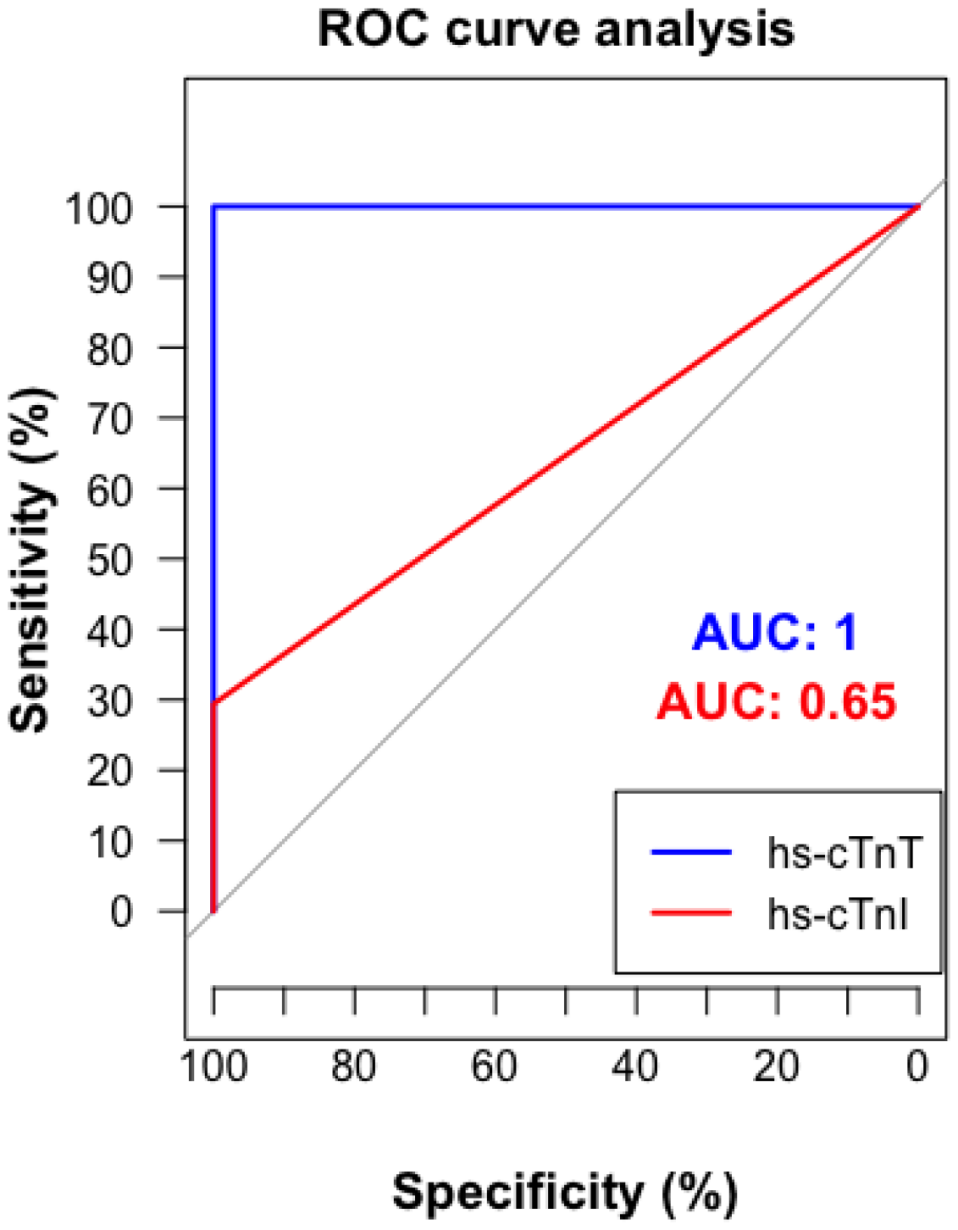
Diagnostic performance of high‐sensitivity cardiac troponin T (hs‐cTnT) and high‐sensitivity cardiac troponin I (hs‐cTnI): receiver operating characteristic (ROC) curve analysis for cardiac involvement. The curve demonstrates sensitivity versus specificity for each biomarker to detect cardiac involvement. The area under the curve (AUC) values are included to quantify the diagnostic accuracy of hs‐cTnT and hs‐cTnI.

Applying the comprehensive definition of cardiac involvement increased the detection rate by 20%, identifying cardiac involvement in 34 of 35 cases (97.1%). Of these, all had an hs‐cTnT level measured at ≥14 ng/L, the recommended 99th percentile upper limit of normal (ULN) [28], whereas 10 (28.6%) had an hs‐cTnI concentration ≥ 45 ng/L, the recommended 99th percentile ULN for Siemens Atellica IM [19]. Details on the diagnostic performance of hs‐cTnT and hs‐cTnI including sensitivities, specificities, positive predictive value, and negative predictive value are listed in Tables 2 and 3. ROC analysis demonstrated an excellent ability of hs‐cTnT to identify cardiac involvement, with an AUC of 1.00 (Figure 3). The AUC was higher for hs‐cTnT than for hs‐cTnI (AUC = 1.0 [SE] vs. 0.65 [SE], *p* = 0.067236).

**TABLE 2:** Diagnostic performance of hs‐cTnT.

	*n* (%)	hs‐cTnT ≥ 14 ng/L, *n* (%)	Sensitivity (95% CI)	Specifity (95% CI)	NPV (95% CI)	PPV (95% CI)
TTE LVEF < 52%	24 (68.6)	24 (100)	100 (86–100)	9.1 (0.2–41)	100	70.1 (67–74)
cMRI LVEF < 52%	8 (22.9)	8 (100)	100 (63–100)	9.1 (0.2–41)	100	44.4 (39–49)
TTE LVEF < 52% and ECG abnormalities	27 (77.1)	27 (100)	100 (87–100)	12.5 (0.3–53)	100	79.4 (75–83)
TTE or cMRI LVEF < 52% and LGE	29 (82.3)	29 (100)	100 (88–100)	16.7 (0.5–72)	100	85.3 (80–89)
TTE or cMRI LVEF < 52% or LGE and ECG abnormalities	30 (85.7)	30 (100)	100 (88–100)	20 (0.5–72)	100	88.2 (83–92)
Cardiac involvement^a^	34 (97.1)	34 (100)	100 (90–100)	100 (3–100)	100	100

Abbreviations: CI, confidence interval; cMRI, cardiac magnetic resonance imaging; ECG, electrocardiogram; hs‐cTnT, high‐sensitivity cardiac troponin T; LGE, late gadolinium enhancement; LVEF, left ventricular ejection fraction; NPV, negative predictive value; PPV, positive predictive value; TTE, transthoracic echocardiography.

^a^ Cardiac involvement requires meeting following criteria: reduced LVEF or presence of LGE on contrast‐enhanced MRI, ventricular morphological abnormalities on TTE or cMRI, diastolic dysfunction evidence, pathological myocardial biopsy findings, abnormal ECG, rhythm disorders, conduction abnormalities, arterial hypertension, or coronary vessel disease. Reduced LVEF is assessed via cMRI or, if not possible, through TTE.

**TABLE 3:** Diagnostic performance of hs‐cTnI.

	*n* (%)	hs‐cTnI ≥ 45 ng/L, *n* (%)	Sensitivity (95% CI)	Specifity (95% CI)	NPV (95% CI)	PPV (95% CI)
TTE LVEF < 52%	24 (68.6)	8 (33)	33.3 (16–55)	81.8 (48–98)	36 (27–46)	80 (50–94)
cMRI LVEF < 52%	8 (22.9)	2 (25)	25 (3–65)	81.8 (48–98)	60 (48–71)	50 (15–85)
TTE LVEF < 52% and ECG abnormalities	27 (77.1)	8 (29.6)	29.6 (14–50)	75 (35–97)	24 (17–36)	80 (51–94)
TTE or cMRI LVEF < 52% and LGE	29 (82.3)	9 (31)	33.3 (16–55)	81.8 (48–98)	36 (27–46)	80 (50–94)
TTE or cMRI LVEF < 52% or LGE and ECG abnormalities	30 (85.7)	9 (30)	30 (15–49)	80 (28–100)	16 (10–24)	90 (59–98)
Cardiac involvement^a^	34 (97.1)	10 (29.4)	29.4 (15–48)	100 (3–100)	4 (3–5)	100

Abbreviations: CI, confidence interval; cMRI, cardiac magnetic resonance imaging; ECG, electrocardiogram; hs‐cTnI, high‐sensitivity cardiac troponin I; LGE, late gadolinium enhancement; LVEF, left ventricular ejection fraction; NPV, negative predictive value; PPV, positive predictive value; TTE, transthoracic echocardiography.

^a^ Cardiac involvement requires meeting following criteria: reduced LVEF or presence of LGE on contrast‐enhanced MRI, ventricular morphological abnormalities on TTE or cMRI, diastolic dysfunction evidence, pathological myocardial biopsy findings, abnormal ECG, rhythm disorders, conduction abnormalities, arterial hypertension, or coronary vessel disease. Reduced LVEF is assessed via cMRI or, if not possible, through TTE.

Neither the diagnostic performance nor the discriminatory ability of hs‐cTnT or hs‐cTnI was tested using sex‐specific 99th percentile ULN cutoffs.

**FIGURE 4:**
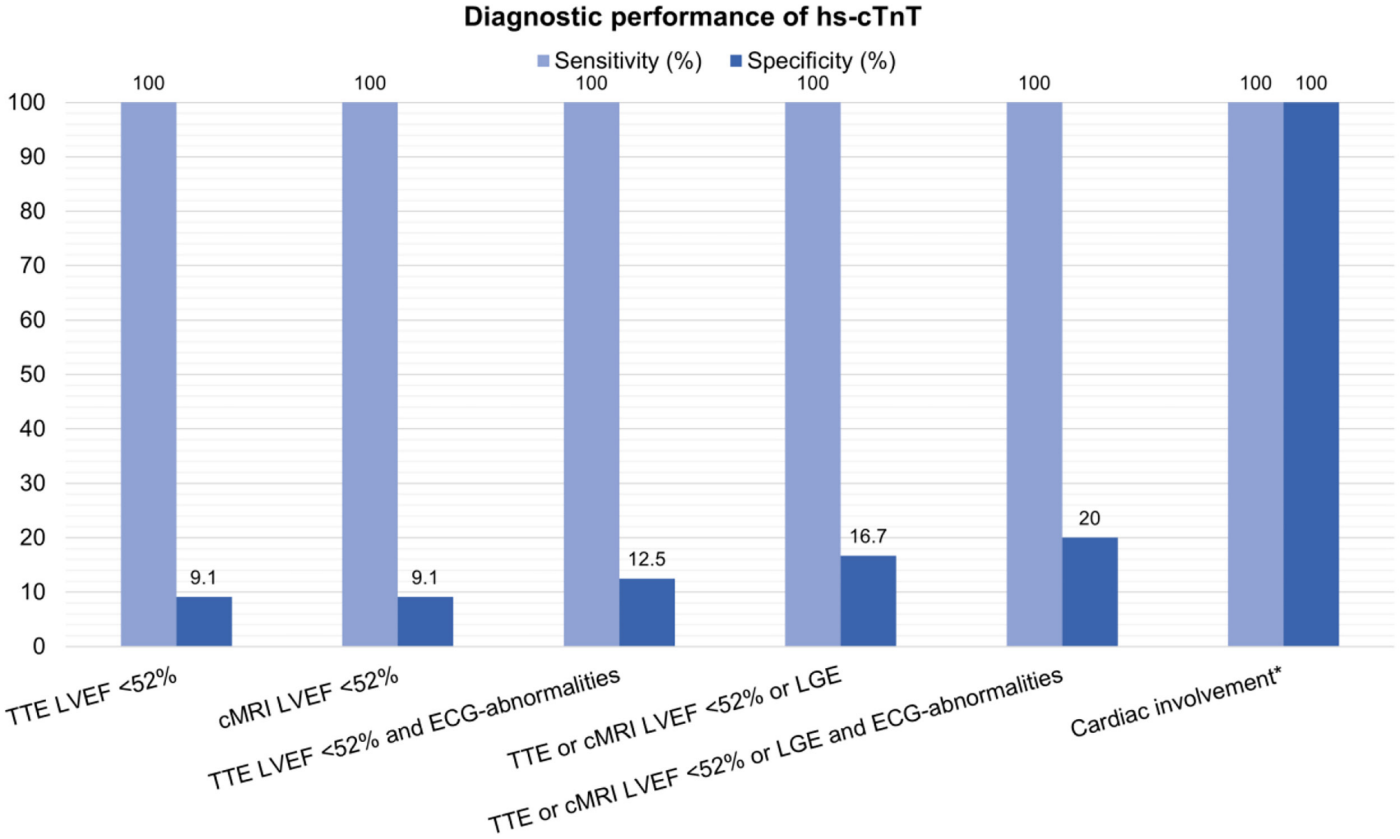
Diagnostic performance of high‐sensitivity cardiac troponin T (hs‐cTnT) by diagnostic tool. cMRI, cardiac magnetic resonance imaging; ECG, electrocardiogram; LGE, late gadolinium enhancement; LVEF, left ventricular ejection fraction; TTE, transthoracic echocardiography. *Cardiac involvement requires meeting the following criteria: reduced LVEF or presence of LGE on contrast‐enhanced MRI, ventricular morphological abnormalities on TTE or cMRI, diastolic dysfunction evidence, pathological myocardial biopsy findings, abnormal ECG, rhythm disorders, conduction abnormalities, arterial hypertension, or coronary vessel disease. Reduced LVEF is assessed via cMRI or, if not possible, through TTE.

**FIGURE 5:**
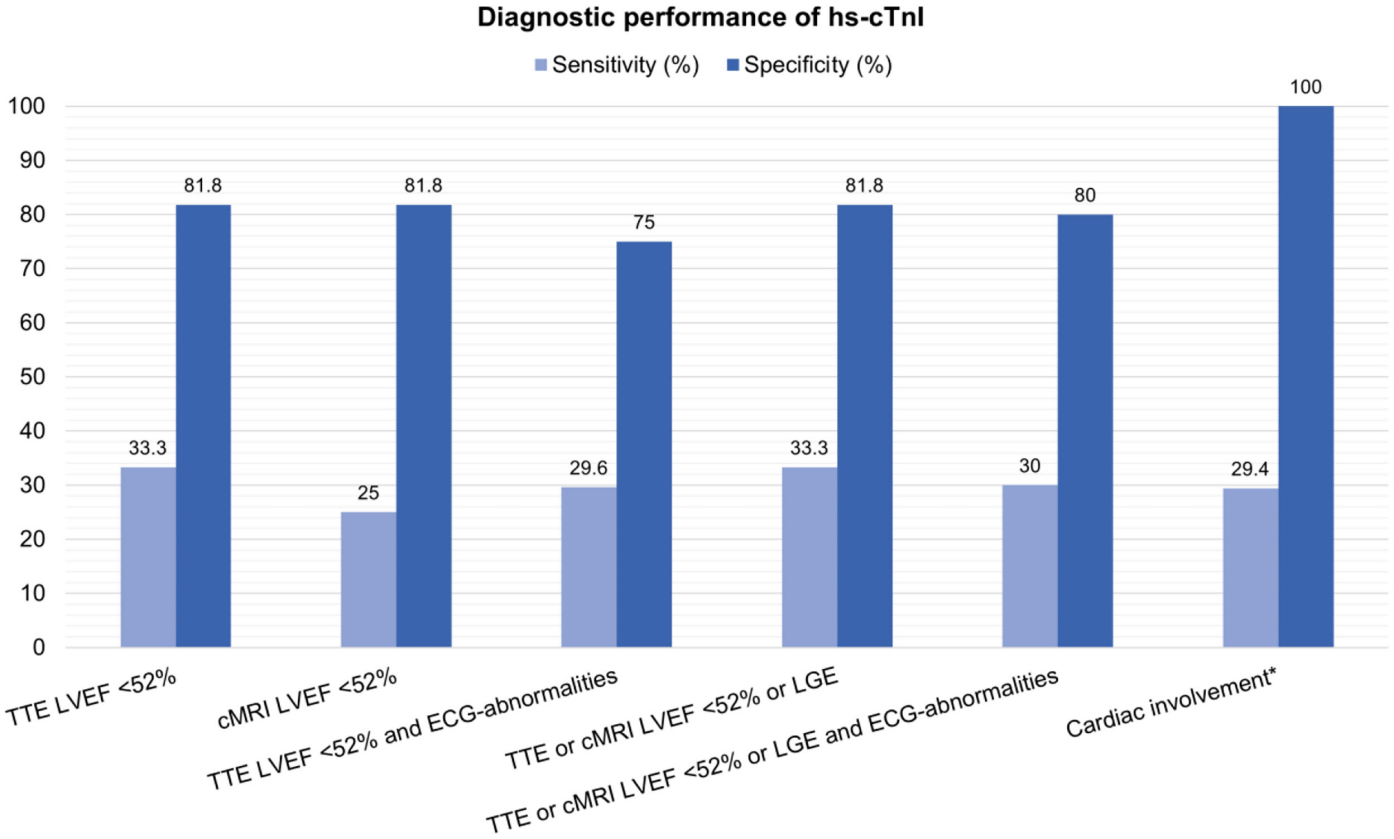
Diagnostic performance of high‐sensitivity cardiac troponin I (hs‐cTnI) by diagnostic tool. cMRI, cardiac magnetic resonance imaging; ECG, electrocardiogram; LGE, late gadolinium enhancement; LVEF, left ventricular ejection fraction; TTE, transthoracic echocardiography. *Cardiac involvement requires meeting the following criteria: reduced LVEF or presence of LGE on contrast‐enhanced MRI, ventricular morphological abnormalities on TTE or cMRI, diastolic dysfunction evidence, pathological myocardial biopsy findings, abnormal ECG, rhythm disorders, conduction abnormalities, arterial hypertension, or coronary vessel disease. Reduced LVEF is assessed via cMRI or, if not possible, through TTE.

Diagnostic performance largely depended on the definition criteria of cardiac involvement. Using myocardial biopsy, TTE or cMRI including LGE, and inclusion of other cardiac manifestations of skeletal muscle dystrophies such as arrhythmias or conduction abnormalities and diastolic dysfunction resulted in the highest clinical specificities for hs‐cTnT (100%, 95% confidence interval [CI] = 3–100) and hs‐cTnI (100%, 95% CI = 3–100; Figures 4 and 5).

**FIGURE 6:**
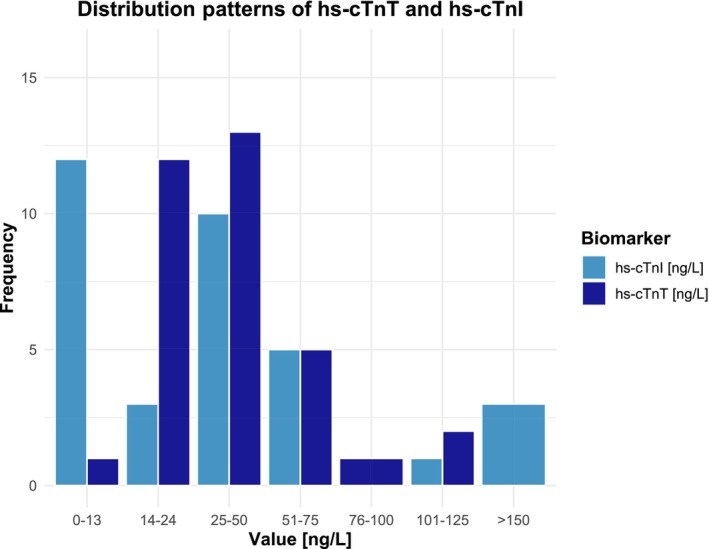
Comparative distribution of high‐sensitivity cardiac troponin T (hs‐cTnT) and high‐sensitivity cardiac troponin I (hs‐cTnI) levels in patients with cardiac involvement.

**FIGURE 7:**
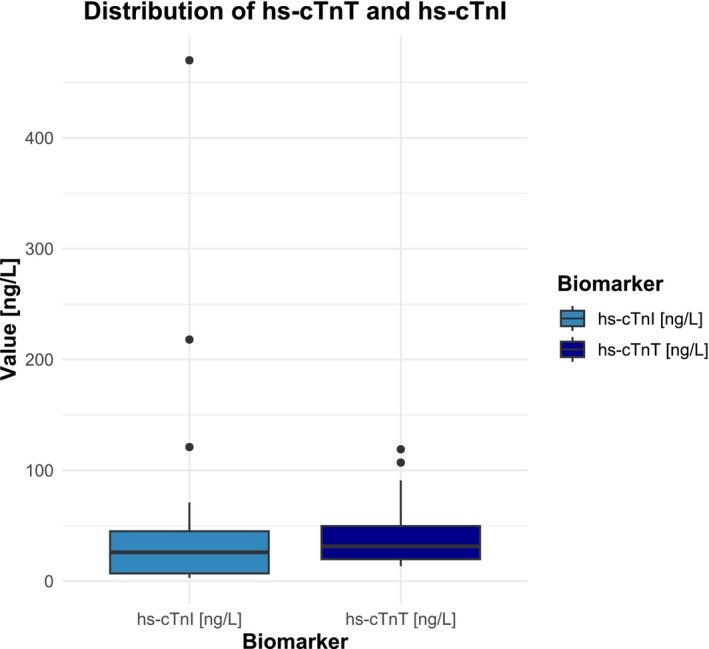
Boxplot provides insights into high‐sensitivity cardiac troponin T (hs‐cTnT) and high‐sensitivity cardiac troponin I (hs‐cTnI) distributions in cardiac involvement. One outlier was excluded from the hs‐cTnI group.

Compared to the criteria to detect cardiac involvement in clinical routine, use of the more comprehensive definition increased numbers of cases with cardiac involvement from 27 (77.1%) to 34 (97.1%). Thus, cardiac involvement was diagnosed in all but one patient (2.9%). This patient exhibited an hs‐cTnT and hs‐cTnI of 7.25 and 19 ng/L, respectively, resulting in a calculated specificity of 100% for both hs‐cTn assays. The distribution patterns of hs‐cTnI and hs‐cTnT in patients with cardiac involvement are visually represented in Figures 6 and 7, respectively.

## DISCUSSION

Using a broad definition, we detected cardiac involvement in virtually every patient with a confirmed skeletal muscle dystrophy in this study. Our findings appear provocative, as prevalence is considerably higher than previously reported. In a large observational study on 211 patients with skeletal muscle disorders [22], prevalence of mild or severe cardiac disease was detected in 49% of cases. That study had included a broad range of muscle disorders including noninflammatory myopathies, neuropathies, myasthenic syndromes, myositis (primary, secondary, and overlap syndromes), autoimmune diseases with muscular symptoms, and muscle symptoms of unknown origin. In contrast, our study included muscle dystrophies in all but one case with myositis. In addition, mild or early cardiac manifestation escaped detection in the study by du Fay de Lavallaz et al. [22], because diagnosis required either the presence of LVEF < 40% (instead of <50%), NT‐pro BNP > 400 ng/L (instead of >125 ng/L), presence of New York Heart Association class ≥ II (instead of any American Heart Association [AHA] stage) [29], presence of severe valvular disease, or a history of previous acute myocardial infarction (AMI).

Contrary to the hypothesis that unexplainable elevations of hs‐cTnT may be attributed to cTn release from chronically damaged skeletal muscle, we rather found a striking underdiagnosis of cardiac involvement in muscle dystrophies using standard imaging methods. We believe that this shortcoming may be overcome by the use of more sensitive imaging methods including strain analysis using 2D TTE or MRI, and assessment of diastolic LV function and RV function. Accordingly, and distinct from previous studies, utilization rates of TTE (100%) and MRI (54%) were higher in our study. In addition, in our study, including occurrence of atrial fibrillation, abnormal blood pressure or hypoxemia, and other potential causes of myocardial injury instead of only focusing on structural and obvious functional abnormalities improved detection of cardiac involvement. Accordingly, cardiac involvement was identified at earlier stages (AHA/American College of Cardiology stage A and B) [29] and was defined more comprehensively in our study compared to many previous studies. Although we cannot exclude a re‐expression and release of hs‐cTnT from skeletal muscle damage, our findings also do not support this hypothesis, at the moment. Our study rather strengthens previous observations that hs‐cTnT or hs‐cTnI represent more sensitive and specific indicators of myocardial damage than 2D TTE and cMRI. The latter may miss early ultrastructural abnormalities or subtle/diffuse fibrosis of myocardial tissue due to the limited spatial resolution [16, 30]. MRI studies have reported a threshold that limits visualization of localized LGE to at least 200 mg of infarcted myocardium [31]. In another experimental study, human myocardium was spiked into human serum [32]. A strong linear correlation between cTnT release and myocardial mass was established, and only 25 mg of the myocardium was required for detection by an hs‐cTnT concentration exceeding the 99th percentile ULN (≥14 ng/L). This and other MRI studies support the claim that hs‐cTn is almost 10 times more sensitive than contrast‐enhanced MRI [33, 34, 35, 36, 37]. Another small MRI study on 136 patients with Duchenne muscular dystrophy with preserved LV function and no overt signs of cardiac involvement used an MRI‐based strain technique, the transmural myocardial strain profile (TMSP), in 136 patients with Duchenne muscular dystrophy [17]. The presence of an abnormal TSMP in 36 patients preceded the echocardiographic identification of new wall motion abnormalities in the posterior wall and a significant reduction of LVEF from a mean of 60% to 53% at 1 year of follow‐up [17].

Overall, we raise the possibility that previous studies systematically underdiagnosed the presence of cardiac involvement in confirmed muscle dystrophies; we found a strong association between the comprehensiveness of the definition criteria for cardiac involvement and the diagnostic performance of hs‐cTn.

Applying this broader definition of cardiac involvement increased numbers of patients with cardiac involvement significantly, thereby reducing numbers of patients with confirmed muscle dystrophies without cardiac involvement. Thus, specificities for the absence of cardiac involvement were 100% for hs‐cTnT and hs‐cTnI. Interestingly, although sensitivities remained 100% for hs‐cTnT, only a modest increase from 25% to 33.3% was noted for hs‐cTnI when the manufacturer‐recommended 99th ULN was used. The observed imbalance may be due to differences in assay performance related to factors such as compartmentalization, release kinetics, epitope variability, or molecular weight. Although this discrepancy has been reported earlier, the reasons remain still unclear. Wildi et al. reported inconsistencies in the diagnosis of AMI due to clinical decision values of several hs‐cTnI assays that were biologically not equivalent [38]. In that study, the biologically equivalent hs‐cTnI value for the Abbott Architect assay corresponding to the clinical decision value for hs‐cTnT was less than half the approved decision value for hs‐cTnI. Therefore, nearly all of reported inconsistencies were related to an underdiagnosis of AMI with hs‐cTnI. It is tempting to speculate that too low decision values also account for the inferior sensitivities for diagnosis of cardiac involvement using the Siemens Atellica hs‐cTnI assay in our study. This hypothesis is further corroborated in another study on 211 patients with skeletal muscle disorders including but not limited to noninflammatory myopathies [22]. In patients with confirmed mild or severe cardiac disease, hs‐cTnT values were consistently above ULN, whereas hs‐cTnI values from three different hs‐cTnI assays (Abbott Architect, Beckman Coulter Access, and Siemens Dimension Vista) were rarely above the approved ULN. Despite adjustments of the 99th percentile (ULN) to a bioequivalent higher level, the issue persisted with two hs‐cTnI assays and was only partially addressed for one (Abbott Architect hs‐cTnI) [22]. Regarding possible explanations, these investigators hypothesize that there might be some degree of preferential release of cTnT versus cTnI from cardiomyocytes attributable to chronic cardiac disease that may have contributed to the observed hs‐cTnT/hs‐cTnI mismatch. But this observation could also suggest that the lower prevalence of elevated hs‐cTnI may result from assay‐specific factors, including differences in epitopes or analytical interference, which could lead to underestimation of myocardial damage sensitivity and prevalence. Additionally, an incorrectly high upper reference limit could contribute to an underestimation of sensitivity and prevalence of pathological myocardial damage [38].

### Limitations

It is important to acknowledge the limitations of our study. First, the relatively small sample size of our cohort increases the possibility of type I error. Second, our study cohort comprised a heterogenous spectrum of the most prevalent muscle dystrophies. However, our cohort does not represent disease prevalence in the general population due to selection and referral bias. This bias is also reflected in the severe imbalance of our dataset, where 97% of the cohort had cardiac involvement after applying the broader definition of cardiac involvement, increasing the risk of overfitting in statistical models. The overrepresentation of cardiac involvement likely results from the referral of patients to our specialized cardiology outpatient clinic, contributing to the higher observed prevalence of cardiac manifestations. In addition, our findings cannot be generalized to other nondystrophic skeletal muscle diseases where cardiac troponin elevations have been reported. Third, upon the application of a broader definition of cardiac involvement compared to previous studies, the number of controls diminished to a single patient. Lastly, although almost every patient with muscle dystrophy met the criteria for cardiac involvement, the prevalence of structural heart disease may have been underdiagnosed in our study due to the limited use of cMRI (~50% utilization rate). We believe that future studies should use MRI instead of 2D TTE as the reference method to detect ultrastructural heart disease, particularly to explain elevated hs‐cTn in patients with skeletal muscle disorders beyond dystrophinopathies.

### Conclusions

In conclusion, our study underscores the superior diagnostic performance of elevated hs‐cTnT over hs‐cTnI in discriminating cardiac involvement among individuals with confirmed skeletal muscle dystrophies, exhibiting very high sensitivity and 100% specificity. Previous discrepancies in diagnostic accuracy may be attributed to the utilization of less sensitive imaging techniques or incomplete evaluations of cardiac involvement. Moving forward, the adoption of more sensitive imaging modalities and comprehensive assessments of cardiac function are imperative to enhance the detection and management of cardiac complications in this patient population.

## AUTHOR CONTRIBUTIONS

**Mustafa Yildirim:** Conceptualization; methodology; software; data curation; formal analysis; validation; investigation; funding acquisition; visualization; writing – review and editing; writing – original draft; project administration. **Christian Salbach:** Visualization. **Christoph Reich:** Visualization. **Regina Pribe‐Wolferts:** Visualization. **Barbara Ruth Milles:** Visualization. **Tobias Täger:** Visualization. **Matthias Mueller‐Hennessen:** Visualization. **Markus Weiler:** Visualization. **Benjamin Meder:** Visualization; validation. **Norbert Frey:** Visualization; validation. **Evangelos Giannitsis:** Conceptualization; methodology; writing – review and editing; validation; visualization; resources; supervision; project administration.

## FUNDING INFORMATION

The study was supported by a research grant from Roche Diagnostics. The sponsor had no influence on the study concept, data collection, or interpretation.

## CONFLICT OF INTEREST STATEMENT

B.R.M. has received research funding from Daiichi Sankyo. E.G. has received honoraria for lectures from Roche Diagnostics, Astra Zeneca, Bayer, Daiichi‐Sankyo, and Lilly Eli Deutschland. He serves as a consultant for Roche Diagnostics, BRAHMS Thermo Fisher Scientific, and Boehringer Ingelheim, and has received research funding from BRAHMS Thermo Fisher Scientific, Roche Diagnostics, Bayer Vital, and Daiichi Sankyo. M.M.‐H. has received research funding from BRAHMS Thermo Fisher Scientific and Roche Diagnostics. N.F. has received speaker honoraria from Daiichi Sankyo, Astra Zeneca, Boehringer Ingelheim, and Bayer Vital. BM was a speaker of the working group for cardiomyopathies of the German Society of Cardiology. He receives speaker honorary and conducts advisory services for HCM‐related therapies. M.W. is a member of the European Reference Network for Neuromuscular Diseases. None of the other authors has any conflict of interest to disclose.

## Data Availability

The data that support the findings of this study are available from the corresponding author upon reasonable request.
